# Molecular epidemiological characteristics of *Mycobacterium leprae* in highly endemic areas of China during the COVID-19 epidemic

**DOI:** 10.3389/fpubh.2024.1148705

**Published:** 2024-01-24

**Authors:** Jiaojiao Zhou, Ziwei Wu, Yi Tong, Santosh Chokkakula, Ying Shi, Haiqin Jiang, Jie Liu, De Wang, Wenyue Zhang, Chen Wang, Tingfang Zhao, Kang Yuan, Tao Li, Lu Ma, Qin Yang, Shizhen Wang, Feng Hong, Hongsheng Wang, Jinlan Li

**Affiliations:** ^1^Key Laboratory of Environmental Pollution and Disease Control, Ministry of Education, School of Public Health, Guizhou Medical University, Guiyang, China; ^2^Guizhou Provincial Center for Disease Control and Prevention, Guiyang, China; ^3^Institute of Dermatology, Chinese Academy of Medical Sciences and Peking Union Medical College, Nanjing, China; ^4^The Fourth Affiliated Hospital of Nanjing Medical University, Nanjing, China; ^5^Jiangsu Key Laboratory of Molecular Biology for Skin Diseases and STIs, National Centre for Leprosy Control, Nanjing, China; ^6^Department of Microbiology, Chungbuk National University College of Medicine and Medical Research Institute, Cheongju, Chungbuk, Republic of Korea; ^7^Center for Global Health, School of Public Health, Nanjing Medical University, Nanjing, China; ^8^Department of Epidemiology and Biostatistics, School of Public Health, Nanjing Medical University, Nanjing, China; ^9^Qianxinan CDC, Qianxinan, China; ^10^Anshun CDC, Anshun, China; ^11^Qiandongnan CDC, Qiandongnan, China; ^12^Guiyang CDC, Guiyang, China; ^13^The Second People’s Hospital of Bijie, Bijie, China

**Keywords:** Leprosy, COVID-19, *Mycobacterium leprae*, Epidemiology, Genotype, strain typing and transmission

## Abstract

**Objectives:**

The present study analyzed the impact of the COVID-19 pandemic on the prevalence and incidence of new leprosy cases, as well as the diversity, distribution, and temporal transmission of *Mycobacterium leprae* strains at the county level in leprae-endemic provinces in Southwest China.

**Methods:**

A total of 219 new leprosy cases during two periods, 2018–2019 and 2020–2021, were compared. We genetically characterized 83 clinical isolates of *M. leprae* in Guizhou using variable number tandem repeats (VNTRs) and single nucleotide polymorphisms (SNPs). The obtained genetic profiles and cluster consequences of *M. leprae* were compared between the two periods.

**Results:**

There was an 18.97% decrease in the number of counties and districts reporting cases. Considering the initial months (January–March) of virus emergence, the number of new cases in 2021 increased by 167% compared to 2020. The number of patients with a delay of >12 months before COVID-19 (63.56%) was significantly higher than that during COVID-19 (48.51%). Eighty-one clinical isolates (97.60%) were positive for all 17 VNTR types, whereas two (2.40%) clinical isolates were positive for 16 VNTR types. The (GTA)9, (TA)18, (TTC)21 and (TA)10 loci showed higher polymorphism than the other loci. The VNTR profile of these clinical isolates generated five clusters, among which the counties where the patients were located were adjacent or relatively close to each other. SNP typing revealed that all clinical isolates possessed the single SNP3K.

**Conclusion:**

COVID-19 may have a negative/imbalanced impact on the prevention and control measures of leprosy, which could be a considerable fact for official health departments. Isolates formed clusters among counties in Guizhou, indicating that the transmission chain remained during the epidemic and was less influenced by COVID-19 preventative policies.

## 1 Introduction

Leprosy, caused by *Mycobacterium leprae* (*M. leprae*), remains a significant public health concern, with more than 200,000 new leprosy cases annually worldwide (1, 2). The number of reported cases per year has remained fairly constant over the past few years, emphasizing that leprosy continues to spread. The distribution and emergence of new leprosy cases are restricted to a small number of countries, with India, Brazil, and Indonesia accounting for more than 80% of cases worldwide, accounting for 59%, 14%, and 9% of cases, respectively (2, 3). This distribution of leprosy was found to be spatially unevenly distributed across countries. Due to the systematic and effective implementation of leprosy eradication programs, leprosy cases in China have declined rapidly over the last few years (4, 5). However, China still reported 422 newly diagnosed cases in 2021, mainly from the southwestern region, such as Sichuan, Hunan, Yunnan, and Guizhou (6–8).

The emergence of severe acute respiratory syndrome coronavirus 2 (SARS-CoV-2) has led to the implementation of policy interventions such as social distancing, population density control, mask-wearing, and other general hygiene improvements. Existing studies suggest that leprosy transmission is primarily due to close contact with leprosy patients, most likely through infectious aerosols produced by coughing and sneezing but also through skin-to-skin contact. We speculate that these intervention strategies during coronavirus disease 2019 (COVID-19) could potentially impact the transmission and distribution of leprosy (1, 9–11). In addition, these policy interventions, as well as social, economic, and health systems, have a strong impact on other diseases, including leprosy, which has been a high prevalence area in Guizhou, China (12). Thus, 127,558 new leprosy cases were detected worldwide in 2020, a decrease of 37% compared to 2019, due to the implementation of the COVID-19 pandemic control programs (13). This is similar to what some authors have said that other public health priorities, such as the COVID-19 pandemic, pose a threat to the sustainability of surveillance and control efforts for diseases such as leprosy from a public health perspective (14, 15).

Recently, multiple-locus variable-number tandem-repeat (VNTR) analysis (MLVA) and single nucleotide polymorphism (SNP) typing have been used in epidemiological investigations to determine genotypic differences between different bacterial species (16–19). As *M. leprae* cannot be cultured on artificial medium, molecular techniques have been used to better characterize the organism (20, 21), including deciphering its genome sequence (22), to determine the exact origin and spread of *M. leprae* (23). Single nucleotide polymorphisms (SNPs) can be used in systematic geographical studies of leprosy (19), and identifying the source of the disease and trace transmission patterns has proven its importance as a valuable framework for global leprosy strain typing (16). Recent studies have reported 4 genotypes and 16 subtypes of *M. leprae* strains worldwide defined by SNPs (19, 23) In addition to SNPs, variable number tandem repeats (VNTRs) were used for genotyping. VNTR-based strain typing is sensitive, and its unique polymorphisms appear to be more suitable for monitoring the spread of *M. leprae* over shorter epidemiological distances (16–18, 24). It can distinguish different leprosy genotypes at the county level and predict the distribution and migration of leprosy (12, 25, 26). It was also observed that the number of some VNTR alleles was correlated with SNP type. Numerous studies have used large panels of VNTR loci and efficiently demonstrated the origin and transmission of leprosy (23, 27–30). Therefore, some scholars have proposed the combination method of VNTR multisite analysis and SNP typing to study leprosy and maximize the role of molecular epidemiology (15).

To better understand the impact of COVID-19 on the transmission of leprosy, we conducted an epidemiological study to assess the causes of measures and behavioral lifestyles associated with COVID-19 that may influence the transmission of leprosy in Guizhou, China. At the same time, we also investigated the genomic characteristics of *M. leprae* strains at the county level in Guizhou, China, at two periods to determine the temporal dynamic nature of leprosy before and during COVID-19.

## 2 Materials and methods

### 2.1 Study designs, enrollment, and data collection

The surveillance data of new leprosy cases in Guizhou from January 1, 2018, to December 31, 2021, were obtained from the Database of China Leprosy Management Information System (LEPMIS). Basic demographic data, including age, sex, source of infection, method of discovery, date of diagnosis, Ridley-Jopling, and WHO classification of patients, were extracted from LEPMIS. Microsoft Excel file (version 2016) was employed to compile data on newly identified leprosy cases and perform chi-square test analysis on their clinical characteristics, such as gender, age, delay, deformity, and disability.

### 2.2 Ethics statement

The present study was approved by the Institutional Review and Ethics Committees of the Institute of Dermatology, Chinese Academy of Medical Sciences, China (2014-KY-003). Tissue samples were collected from all patients after informed consent was obtained.

### 2.3 Sample collection and isolation of *Mycobacterium leprae* genomic DNA from skin biopsies

Skin biopsy samples were collected from all enrolled new leprosy patients at the Guizhou Provincial Center for Disease Control and Prevention from 2018 to 2021. As per the WHO guidelines, the confirmation of leprosy was accomplished by routine skin smear and histopathological examination (31). County-level registration and clinical information on cases in Guizhou are recorded in Supplementary Table S1. The biopsy samples were collected from all confirmed cases of leprosy in 70% ethanol and then transported into the central laboratory facility at the National Center for Leprosy Control, China CDC. The biopsy tissue samples were washed twice with phosphate-buffered saline (PBS) followed by grinding with a glass Dounce homogenizer. Total genomic DNA was isolated from ethanol-fixed biopsy samples by a DNeasy Blood & Tissue Kit (Qiagen, Germany, cat No. 69504) according to the manufacturer’s instructions with minor modifications. The isolated genomic DNA was immediately used for genotyping analysis or stored at-70°C for further use.

### 2.4 Multiplex PCR amplification of VNTR loci

VNTR analysis of *M. leprae* was performed using 17 mini and microsatellite VNTR loci, such as (AC)8b, (GTA)9, (GGT)5, (AT)17, rpoT, 21–3, (AC)9, (AT)15, (AC)8a, 27–5, 6–7, (TA)18, (TTC)21, 18–8, 12–5, 23–3 and (TA)10. Primers for amplification of VNTR loci are listed in Supplementary Table S2 (3). Multiplex PCR was performed with four multiplex PCR combinations in a 20 μL reaction volume containing 12 μL Qiagen^®^ Multiple PCR Mix, 2 μL (2 μM) each primer mix, 2 μL Q solution, and 2 μL DNA template. PCR amplification (Bio-RAD) was performed under the following cyclic conditions: initial denaturation at 95°C for 15 min; 40 cycles of final denaturation at 94°C for 30 s, annealing at 60°C for 90 s, and extension at 72°C for 90 s; and a final step of 72°C for 10 min. After verification by quality check on 2% agarose, the amplified multiplex PCR products were subjected to fragment length analysis (Applied Biosystems 3,130, United States), and the allelic copy number of each VNTR locus was calculated using Peak Scanner software (Applied Biosystems, ver. 1.0, United States).

### 2.5 SNP typing by PCR-RFLP

The SNP loci of *M. leprae* at positions 14,676 (L1), 164,275 (L2), and 2,935,685 (L3) were amplified using previously published primers that can be used to differentiate SNP types (1–4) by RFLP digestion (19, 22) (Supplementary Figure S1, Table S3).

These regions can be amplified with the following reaction ingredients in a final volume of 50 μL: 25 μL of GoTaq^®^ Green Master Mix, 2 μL (10 pmol) of each primer mix, 5 μL of DNA template, and 16 μL of ddH2O. The thermocycling conditions were as follows: initial denaturation at 94°C for 5 min; 45 cycles of denaturation at 94°C for 60 s, annealing at 55°C for 60 s, and extension at 72°C for 2 min; and a final elongation step at 72°C for 10 min. Products that were not SNP typed by the RFLP method were subjected to direct DNA sequencing.

SNP subtyping of *M. leprae* was performed by amplification of the regions using previously published primer sequences as follows: SNP subtypes 1 A-D were identified by sequencing SNPs at positions 8,453, 313,361 and 61,425 (Supplementary Figure S2A, Table S3); SNP 3 K subtypes were determined by first sequencing SNPs at positions 2,312,059 and 413,902; and direct sequencing at positions 2,312,059, 413,902, 1,133,942 and 20,910 aided in the evaluation of other SNP 3 subtypes using information obtained from 3 K subtypes (Supplementary Figure S2B, Table S3) (16, 23).

### 2.6 VNTR copy number and clustering analysis

Clustering was defined based on a comparison of the copy number of VNTRs, considering VNTRs with the same copy number in all 13 alleles, excluding the four most variable loci (32). The unweighted pair group method using category similarity coefficients and arithmetic mean (UPGMA) was used to generate the similarity matrix for the clustering analysis, and then the minimum spanning tree (MST) was constructed using the ggnetwork and ggtree packages in R. The Microsatellite Tool kit (accessed on 27 March 2022^1^; University of the Basque Country, Spain) was used to calculate allele frequency (discriminatory power). The Hunter Gaston Discrimination Index (HGDI) was used for the interpretation of allelic variation (25).

## 3 Results

### 3.1 Epidemiological situation and sampling and data

From 2018 to 2021, 219 new leprosy cases were reported in Guizhou, among which 83 samples were collected from 9 prefectures and 50 counties, including 4, 40, 20 and 19 leprosy patients in 2018, 2019, 2020 and 2021, respectively. Details of registered cases and their clinical information at the county level are recorded in Supplementary Table S2. Although these counties are within the same geographical area, most of them are relatively distant from each other.

**Figure 1:**
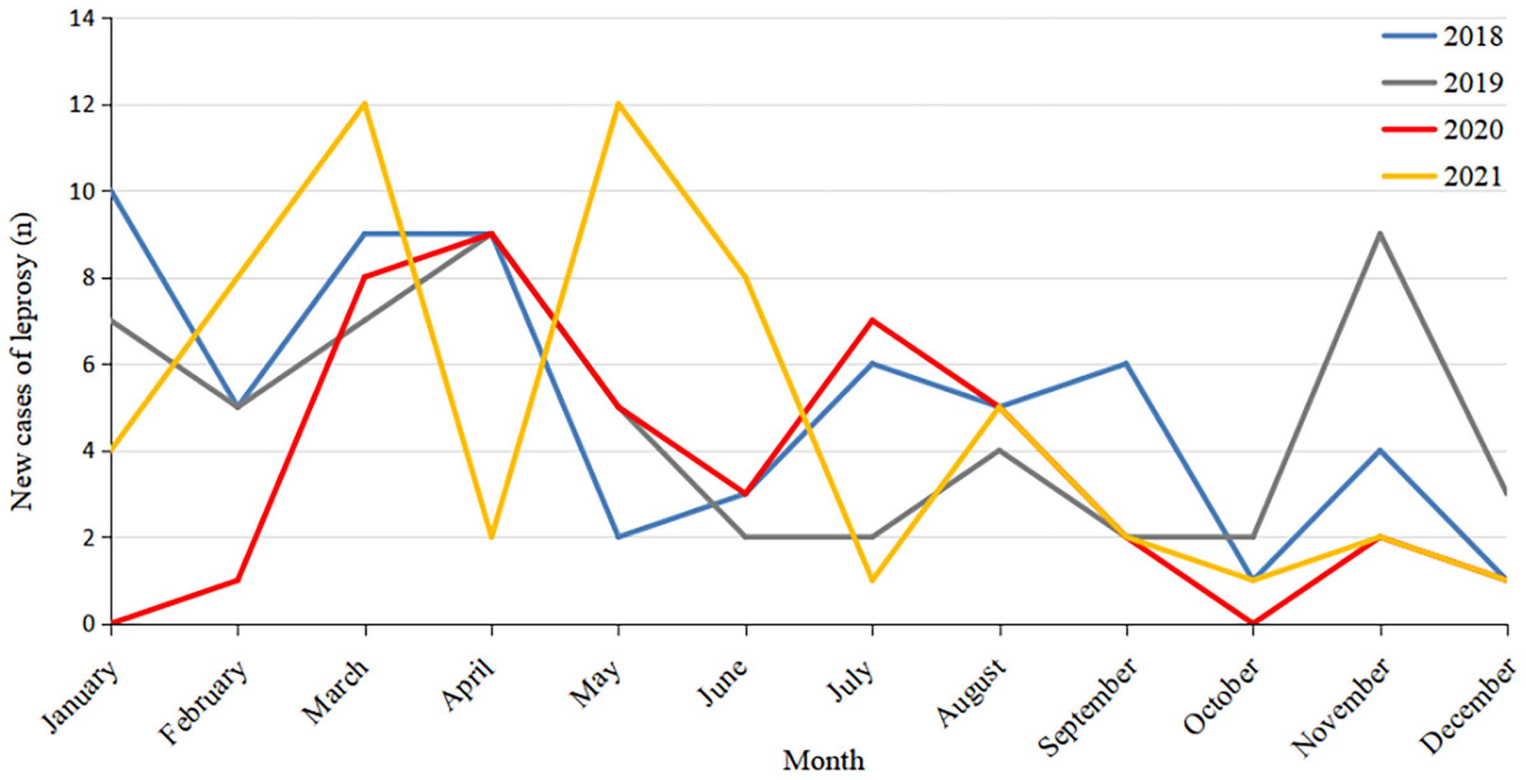
Temporal distribution of new leprosy cases in Guizhou from 2018 to 2021.

Of the 219 new leprosy cases, four were children aged 0 to 14 years, accounting for 1.83% of the total cases. The new leprosy cases were mainly male (66.66%), ethnic minorities (53.42%), farmers (67.12%), and 15–49 years old (77.17%; Table 1). The average annual detection rate was 0.1512/100000.43 cases of leprosy detected in 2020, accounting for 19.63% of the total cases. Considering only the COVID-19 epidemic period (January–March) in Guizhou, the number of new cases in 2021 increased by 167% compared to 2020 (24 confirmed cases in 2021 and 9 in 2020). This suggests that COVID-19 may have had an impact on the detection of leprosy cases (Figure 1).

**Table 1:** Characteristics of new leprosy cases before and during the epidemic of COVID-19.

Variable	Subgroup	2018–2019 [n (%)]	2020–2021 [n (%)]	χ^2^	*p*
Age				4.222	0.123
	≤14 years	1 (0.85)	3 (2.97)		
	15 ~ 49 years	97 (82.20)	72 (71.29)		
	≥50 years	20 (16.95)	26 (25.74)		
Gender				1.112	0.292
	Male	75 (63.56)	71 (70.30)		
	Female	43 (36.44)	30 (29.70)		
Ethnic group				0.080	0.777
	Han Chinese	56 (47.46)	46 (45.54)		
	Minority group	62 (52.54)	55 (54.46)		
Occupation				1.622	0.197
	Farmer	84 (71.19)	63 (62.38)		
	Other	34 (28.81)	38 (37.62)		
Source of infection				3.232	0.199
	Unknown source of infection	66 (55.93)	46 (45.55)		
	Source of infection in the home	33 (27.97)	30 (29.70)		
	Source of infection out of home	19 (16.10)	25 (24.75)		
Detection method				10.716	0.013
	Suspect survey	33 (27.97)	12 (11.88)		
	Household examination	9 (7.63)	10 (9.90)		
	Spot survey	0 (0.00)	0 (0.00)		
	General skin clinic	53 (44.91)	63 (62.38)		
	self-reported	23 (19.49)	16 (15.84)		
Delay in diagnosis				5.015	0.025
	≤12 months	43 (36.44)	52 (51.49)		
	>12 months	75 (63.56)	49 (48.51)		
Type				2.537	0.111
	MB	99 (83.90)	76 (75.25)		
	PB	19 (16.10)	25 (24.75)		
Leprosy reaction				0.720	0.396
	Yes	12 (10.16)	7 (6.93)		
	No	106 (89.83)	94 (93.07)		
Disability grade				0.178	0.673
	G2D	20 (16.95)	15 (14.85)		
	0/1 grade	98 (83.05)	86 (85.15)		
Patient flow				13.754	<0.001
	From out of the province	15 (12.71)	34 (33.66)		
	Cases in the province	103 (87.29)	67 (66.34)		
Total		118	101		

### 3.2 Analysis of case characteristics

**Figure 2:**
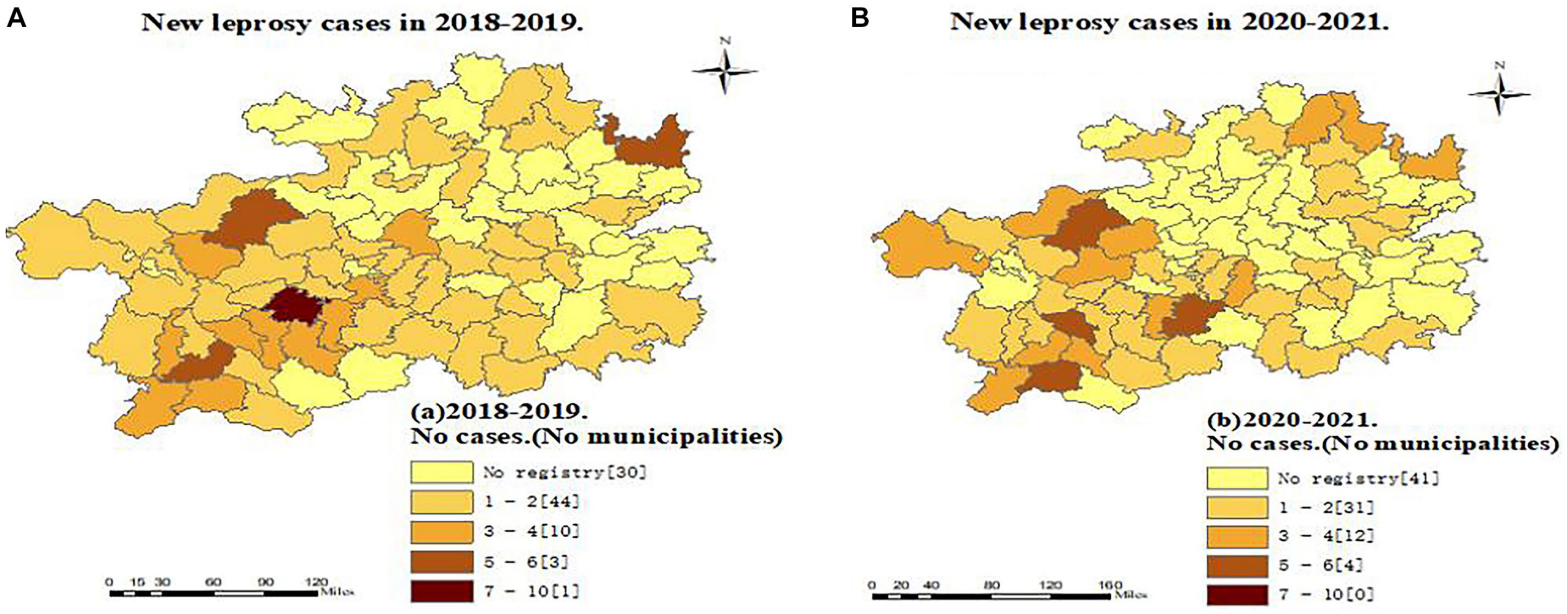
Regional distribution of new leprosy cases in Guizhou before and during the epidemic of the Covid-19.

Comparing the two periods analyzed, we found a reduction in the number of counties reporting leprosy cases: 58 counties in 2018–2019 and 47 in 2020–2021, representing a reduction of 18.97%. Anshun and Qianxian in Guizhou were the regions with the most registered cases in 2018–2019 (23 and 22 cases, respectively), whereas there were 9 cases (−60.87%) and 20 cases (−9.09%), respectively, in 2020–2021 (Figure 2).

The source of infection of 219 new leprosy cases was mostly unknown (51.14%), and the proportion of unknown sources of transmission during COVID-19 was less than that before. Dermatology screening was predominant (52.97%), and the number of new leprosy cases through dermatology clinics during COVID-19 was significantly higher than before (χ^2^ = 12.70, *p* < 0.05); the type of composition was dominated by multibacillary (MB; 79.91%), and the proportion of MB cases composition during COVID-19 was 75.25%, lower than before (83.90%). Of the 219 new leprosy cases, 8.68% had leprosy reactions, and 75.67% had nerve damage (Table 1).

The mean delay between onset and diagnosis was calculated in months. In 2018–2019, the longest delay was 156 months, and the shortest delay was 0.23 months. From 2020 to 2021, the longest delay was 139 months, and the shortest delay was 0.07 months. The number of cases in 2020–2021 with a delay of >12 months was significantly lower than that in 2018–2019 (χ^2^ = 5.02, *p* < 0.05), and 15.98% (35/219) of new leprosy cases had grade 2 deformity (G2D) at diagnosis. The rate of G2D deformity was the highest in 2018 (21.05%) and the lowest in 2020 (6.98%). G2D deformity decreased from 16.95% (2018–2019) to 14.85% (2020–2021). During the COVID-19 outbreak, the number of outflow cases was significantly higher than before (χ^2^ = 13.75, *p* < 0.01; Table 1).

### 3.3 VNTR analysis of *Mycobacterium leprae* strains

Eighty-one clinical isolates (97.6%) were characterized by 17 VNTRs, and two (2.40%) were characterized by 16 VNTRs (Supplementary Figure S3). Sixteen samples from Puding and Baiyun counties failed VNTR typing for the locus GTA9. Among all VNTRs, the (GTA)9, (AT)17, (TA)18, (AT)15, (AC)8a, 6–7, (TTC)21, and (TA)10 loci were found to be highly variable, with an HGDI above 0.6; (AC)8b and (AC)9 were found to be moderately variable (HGDI 0.3–0.6); and the remaining (GGT)5, rpoT, 21–3, 23–3, 18–8, 12–5, and 27–5 loci were reported to be less variable (HGDI <0.3). The allelic copy number and HGDI for each locus are presented in Supplementary Table S4. In analyzing the VNTR profile within and between counties, we observed several distinct strain types with varied county-level geographic distributions. In addition to the common VNTR copy numbers observed in Guizhou, we also observed some specific copy numbers, including 10 and 11 for the loci (AC)8b from the isolates of Puan and Yunyan counties, respectively, and 11 for the loci 6–7 from the isolates of Xing County. The common VNTR copy number of the (GGT)5 loci was 4, and copy numbers 5, 3, and 3 of the (GGT)5 loci were observed in Xixiu, Baiyun, and Dushan counties, respectively. In addition, except for individual counties, the 3-copy number of rpoT, the 2-copy number of 21–3, the 3-copy number of 12–5, and the 2-copy number of 23–3 were predominant in Guizhou.

### 3.4 SNP distribution at the county level

The samples were amplified using primers at locus 3, and 180 bp amplicons were obtained. Restriction digestion of these samples was performed in locus-3, and we found a restriction digestion pattern with 148 bp and 32 bp fragments with nucleotide position C at site 3. These samples were then amplified for locus-1, which yielded a 194 bp amplicon. After restriction of the locus-1 amplicon, all samples remained undigested. All 83 new samples were SNP type-3. All samples were further subtyped. All samples from patients were observed to be subtype K (Supplementary Figure S3).

### 3.5 Population structure and clustering patterns

**Figure 3:**
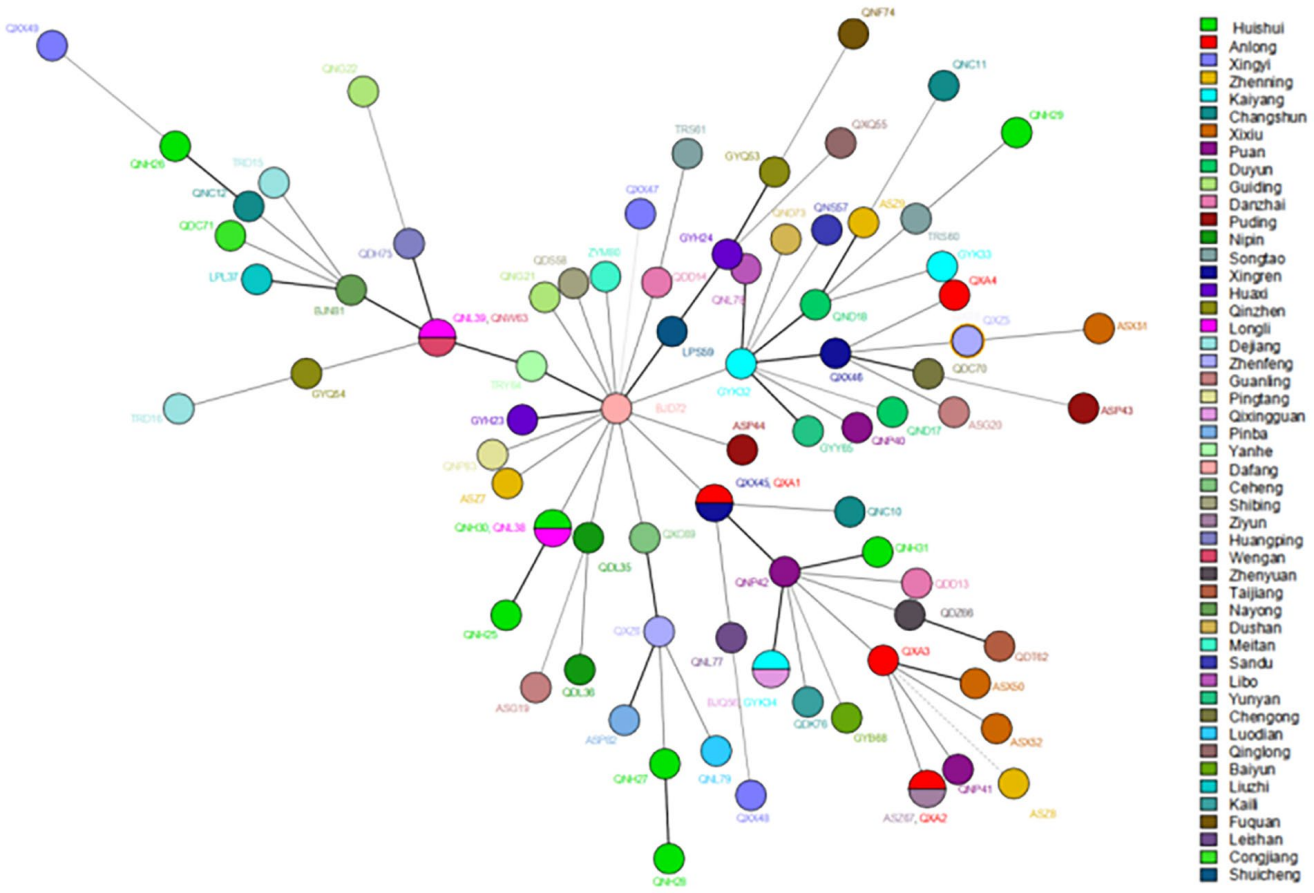
The MST of *Mycobacterium leprae* at the county levels. The circle with divisions designates strains having identical strain types and forming clusters at county levels. Different colors represent different country names.

Regarding cluster analysis, when excluding the four markers with HGDI >0.80 [(GTA)9, (TA)18, (TTC)21, and (TA)10], 78 different genotypes were detected, 73 singletons and five clusters of two patients each, resulting in an overall cluster level of 12.80% (10/78). Three cluster cases are located in the same prefecture-level city in Figure 3; for example, Huishui and Longli counties are adjoined; Longli and Wengan counties are situated relatively close to each other; Xingren and Anlong counties are adjoined. The other two clusters were located in Anlong and Ziyun counties and Kaiyang and Qixingguan counties, which are geographically far apart. It is worth noting that all strains from these clusters reported similar SNP 3 K types, further demonstrating the genetic similarity at the county level.

### 3.6 Genotype comparison before and during the COVID-19 epidemic

**Figure 4:**
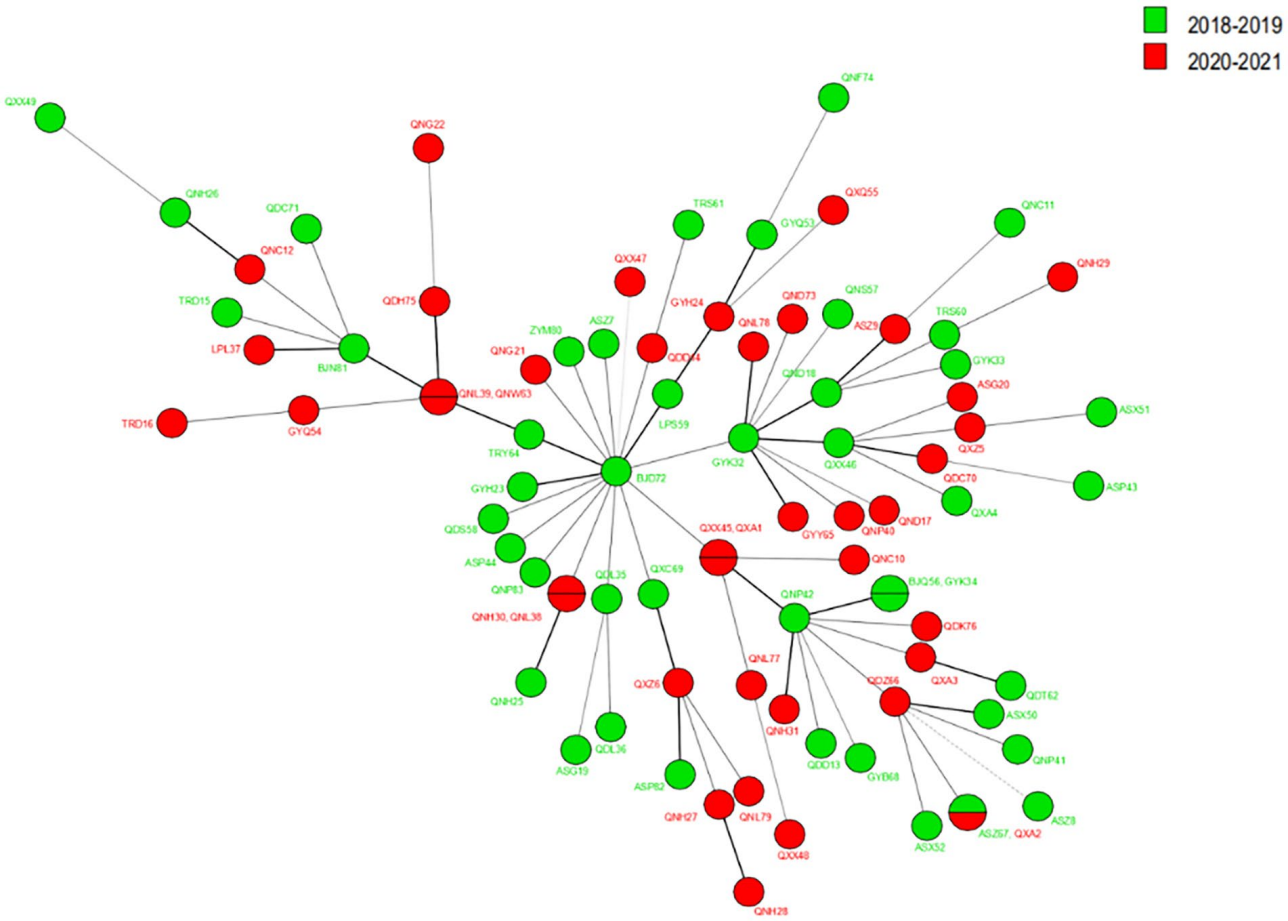
The MST of *M. leprae* isolates from Guizhou in 2018–2019 and 2020–2021.

The VNTR genotyping pattern of *M. leprae* isolates from Guizhou was analyzed before (2018–2019) and during the (2020–2021) epidemic of COVID-19. Regarding the cluster analysis, when the cluster analysis included 13 markers, we observed that the genotype remained relatively stable during these periods, as shown in Figure 4; however, some of the loci slightly varied from the others. As a stable VNTR pattern, the same SNP type (SNP 3 K) was found in all new cases, further supporting genome integrity during this period. Meanwhile, we observed less variation in branching patterns and less variation in strains of *M. leprae*, indicating that the transmission chain of leprosy still exists and that the transmission pattern is less affected by the prevention and control policy during the COVID-19 epidemic.

## 4 Discussion

The World Health Organization is effectively implementing leprosy control programs toward the elimination of leprosy across the globe, but the last stone has not been shaken (26, 33). China still has several provinces with leprosy epidemics that are unevenly distributed (7, 34). Meanwhile, COVID-19 and public health measures have taken place, such as lockdown measures and restrictions on the migration and travel of people, which have created barriers to access to leprosy services, such as difficulties in accessing care centers and reduced or closed service centers (9, 35). Therefore, we analyzed the distribution, transmission, and incidence of leprosy before and during COVID-19 through statistical analysis and molecular epidemiological approaches.

The survey report showed that the number of leprosy cases in Guizhou showed a downward trend from 2018 to 2021, and the number of leprosy cases in 2020 was much lower than that in the other 3 years. Meanwhile, in other years, new cases of leprosy began to be detected in January, but in the first few months of 2020, fewer cases were detected than in other years, with the peak of cases moving backward. Moreover, the number of leprosy cases in 2021 showed a rebound trend, especially when compared with the period of COVID-19 in early 2020 (1–3 months). These results suggest that due to the government’s policies on COVID-19 prevention and control, some people’s mobility is restricted by COVID-19-related factors, leading to difficulties in accessing medical services. At the same time, the Centers for Disease Control and Prevention and medical departments have focused on work related to COVID-19, reducing the proactive screening and skin examination of the public, which has affected the early detection and diagnosis of patients and resulted in a significant reduction in the number of reported cases.

The observed decrease in the number of counties registering new cases of leprosy in Guizhou may indicate that the COVID-19 pandemic has had a dramatic impact on the health care system in all counties in Guizhou, as well as a major setback in the prevention and control of leprosy. In the context of the COVID-19 pandemic, many national and international societies of Dermatology have proposed to discourage or postpone non-emergency consultations and hospitalizations (35, 36), resulting in a loss of business for leprosy surveillance programs and limiting access to hospital services for people with leprosy. At the same time, people living in some counties in Guizhou Province are at greater risk of COVID-19 infection due to the lack of proper sanitation facilities and limited medical infrastructure. Every year, a large number of the floating population from Guizhou go to economically developed eastern provinces such as Zhejiang to work as migrant workers (37, 38). The present study found that during COVID-19, the number of returning cases in the province was much higher than that before the outbreak and that mobile patients and most provincial hospitals had no experience in detecting leprosy. It can be assumed that they had time and opportunity to visit the local CDC or hospital for diagnosis because they were stranded in Guizhou due to the COVID-19 epidemic. It is necessary to strengthen the early detection and standardized management of leprosy cases in the floating population. Guizhou has one of the highest rates of leprosy in China (7, 8, 26), and local dermatologists and professionals from disease control and prevention agencies have been trained in leprosy diagnosis and treatment for many years. Greater vigilance in leprosy allows active and conscious identification and diagnosis of leprosy, leading to timely detection of patients, especially in low endemic areas, which is valuable for early detection and helps to reduce the rate of malformations.

This study showed that the highest number of dermatologic patients was seen before and during the COVID-19 epidemic, suggesting that enhanced training of dermatologists in healthcare facilities and increased capacity and vigilance in the province’s dermatologic leprosy surveillance network for suspicious symptoms of leprosy could be an important tool for detecting leprosy cases in low endemic states (39). At the same time, close contact is an important means of transmission of leprosy (11). This study found close contact with pediatric cases in Guizhou in recent years by examination. Therefore, regular examination of close contact with patients should still be carried out in areas with a low prevalence of leprosy. Suspicious reports of leprosy were mainly provided by rural and village doctors. This survey showed that the number of patients found by clue investigation decreased significantly during the epidemic of COVID-19, which may be related to the impact on the work of township health centers and village doctors during the epidemic of COVID-19. Among the self-reported patients, 47.22% of the source of infection was unknown, suggesting that the public should strengthen the publicity of leprosy knowledge and improve self-awareness.

The main source of leprosy infection is MB cases. The proportion of MB cases among leprosy patients in Guizhou was 83.90% in 2018–2019 and 75.25% in 2020–2021, MB cases accounted for the largest proportion both before and during COVID-19, which reminds us to pay attention to the early detection and treatment of MB cases to prevent further spread of leprosy. According to available studies, the hidden prevalence of leprosy is high (40, 41). In this study, we observed that the G2D and delay period of leprosy cases during COVID-19 were lower than those before COVID-19, which may be related to the improvement in people’s health awareness, and the specific reasons need further investigation.

According to existing genotyping studies of leprosy strains, the predominant SNP types in China are 3 K and 1D, especially SNP 3 K (16, 42), which is consistent with the results of the present study. However, SNP 1D was not found in our study, which was evidence that the transmission efficiency of leprosy in Guizhou was low, and the transmission power of other subtypes except SNP 3 K was in a state of attenuation. In addition, analysis of VNTR loci showed that the isolates in this study were similar to previously published data in China, with slight differences (12, 42, 43). The isolates formed clusters among counties in Guizhou, indicating that the transmission chain still exists and that the disease is still spreading. In addition to the county-level distribution, we found two clusters, both of which consisted of two distant counties. Additionally, we discovered that there was no migration of cases. Therefore, we believe that these cases may have been acquired through other transmission routes; for example, interprovincial transmission of leprosy was more common in the past (43).

Meanwhile, a comparison of the leprosy clustering patterns before and during COVID-19 shows that the leprosy transmission chain still exists and is less affected by policies during the COVID-19 epidemic.

This study has several limitations. First, fewer new cases were enrolled in 2020–2021, which may have masked transmission links caused by factors other than patient contact. Second, leprosy has a long incubation period, and the impact of the strategy may lag. Last, data on COVID-19 leprosy cases may not be accurately reported; for instance, there might be registration delays altering the diagnosis date. Therefore, it is necessary to include future new cases for comparison to further confirm whether the prevention and control strategies of COVID-19 have indirect effects on the distribution and migration of leprosy.

In conclusion, although there is a low prevalence of leprosy in Guizhou, effective public health information campaigns are still needed to consistently raise awareness of leprosy family health education and eliminate public fear of leprosy, especially to continue to strengthen the capacity of the provincial leprosy surveillance network. At the same time, relevant measures of leprosy prevention and control should be implemented, and screening work can be performed in key areas and populations to achieve early detection and treatment for leprosy patients to effectively eliminate the transmission of leprosy. The current molecular epidemiological study explicated the distribution and migration status of leprosy in Guizhou, China. The clustering pattern was lower at the county level in Guizhou. However, the isolates were distributed in small clusters among the counties, suggesting that leprosy transmission still exists. This suggests that there is demand for better approaches to further prevent the ongoing transmission of leprosy at the county level through close contact screening.

## Data availability statement

The datasets presented in this study can be found in online repositories. The names of the repository/repositories and accession number(s) can be found at: https://www.ncbi.nlm.nih.gov/ PRJNA957589.

## Ethics statement

The studies involving humans were approved by The Institutional Review and Ethics Committees of the Institute of Dermatology, Chinese Academy of Medical Sciences, China (2014-KY-003). The studies were conducted in accordance with the local legislation and institutional requirements. The human samples used in this study were acquired from primarily isolated as part of your previous study for which ethical approval was obtained. Written informed consent for participation was not required from the participants or the participants’ legal guardians/next of kin in accordance with the national legislation and institutional requirements.

## Author contributions

JinL and HW were responsible for the experimental design and review of the manuscript. SC, JZ, and ZW performed the laboratory work, data analysis, and drafting of the manuscript. YT, JieL, DW, CW, TZ, KY, TL, LM, QY, and SW participated in data and sample collection. FH, WZ, YS, and HJ critically reviewed the manuscript. All authors have reviewed the manuscript, contributed to the article, and approved the submitted version.

## Footnotes

^1^ http://insilico.ehu.es/mini_tools/discriminatory_power/index.php
