# Supplementary material for: Molecular epidemiological characteristics of Mycobacterium leprae in highly endemic areas of China during the COVID-19 epidemic
Source: Front Public Health. 2024 Jan 24;12:1148705. doi: 10.3389/fpubh.2024.1148705 (PMC10847240; doi:10.3389/fpubh.2024.1148705)

Table S1. Clinical information registration of leprosy cases in Guizhou，2018-2021

| ID | Confirmed time | Gender | City | County |
| --- | --- | --- | --- | --- |
| QXA1 | 2020 | male | Qianxinan | Anlong |
| QXA2 | 2020 | female | Qianxinan | Anlong |
| QXA3 | 2020 | male | Qianxinan | Anlong |
| QXA4 | 2019 | male | Qianxinan | Anlong |
| QXZ5 | 2020 | female | Qianxinan | Zhenfeng |
| QXZ6 | 2020 | female | Qianxinan | Zhenfeng |
| ASZ7 | 2019 | female | Anshun | Zhenning |
| ASZ8 | 2019 | female | Anshun | Zhenning |
| ASZ9 | 2020 | male | Anshun | Zhenning |
| QNC10 | 2021 | female | Qiannan | Changshun |
| QNC11 | 2019 | female | Qiannan | Changshun |
| QNC12 | 2020 | male | Qiannan | Changshun |
| QDD13 | 2019 | male | Qiandongnan | Danzhai |
| QDD14 | 2020 | male | Qiandongnan | Danzhai |
| TRD15 | 2019 | male | Tongren | Dejiang |
| TRD16 | 2020 | male | Tongren | Dejiang |
| QND17 | 2021 | male | Qiannan | Duyun |
| QND18 | 2019 | male | Qiannan | Duyun |
| ASG19 | 2019 | male | Anshun | Guanling |
| ASG20 | 2020 | female | Anshun | Guanling |
| QNG21 | 2021 | male | Qiannan | Guiding |
| QNG22 | 2021 | male | Qiannan | Guiding |
| GYH23 | 2019 | female | Guiyang | Huaxi |
| GYH24 | 2020 | male | Guiyang | Huaxi |
| QNH25 | 2019 | female | Qiannan | Huishui |
| QNH26 | 2019 | male | Qiannan | Huishui |
| QNH27 | 2021 | female | Qiannan | Huishui |
| QNH28 | 2021 | female | Qiannan | Huishui |
| QNH29 | 2021 | female | Qiannan | Huishui |
| QNH30 | 2021 | female | Qiannan | Huishui |
| QNH31 | 2021 | female | Qiannan | Huishui |
| GYK32 | 2019 | male | Guiyang | Kaiyang |
| GYK33 | 2018 | female | Guiyang | Kaiyang |
| GYK34 | 2018 | male | Guiyang | Kaiyang |
| QDL35 | 2019 | female | Qiandongnan | Liping |
| QDL36 | 2018 | male | Qiandongnan | Liping |
| LPL37 | 2021 | male | Liupanshui | Liuzhi |
| QNL38 | 2021 | male | Qiannan | Longli |
| QNL39 | 2021 | male | Qiannan | Longli |
| QNP40 | 2020 | female | Qianxinan | Puan |
| QNP41 | 2019 | male | Qianxinan | Puan |
| QNP42 | 2019 | male | Qianxinan | Puan |
| ASP43 | 2019 | female | Anshun | Puding |
| ASP44 | 2019 | female | Anshun | Puding |
| QXX45 | 2021 | female | Qianxinan | Xingren |
| QXX46 | 2019 | male | Qianxinan | Xingren |
| QXX47 | 2020 | female | Qianxinan | Xingyi |
| QXX48 | 2020 | female | Qianxinan | Xingyi |
| QXX49 | 2019 | male | Qianxinan | Xingyi |
| ASX50 | 2019 | female | Anshun | Xixiu |
| ASX51 | 2019 | male | Anshun | Xixiu |
| ASX52 | 2019 | male | Anshun | Xixiu |
| GYQ53 | 2019 | male | Guiyang | Qingzhen |
| GYQ54 | 2020 | male | Guiyang | Qingzhen |
| QXQ55 | 2020 | male | Qianxinan | Qinglong |
| BJQ56 | 2019 | female | Bijie | Qixingguan |
| QNS57 | 2019 | female | Qiannan | Sandu |
| QDS58 | 2019 | female | Qiandongnan | Shibin |
| LPS59 | 2019 | male | Liupanshui | Shuicheng |
| TRS60 | 2019 | male | Tongren | Songtao |
| TRS61 | 2019 | male | Tongren | Songtao |
| QDT62 | 2019 | male | Qiandongnan | Taijiang |
| QNW63 | 2021 | female | Qiannan | Wengan |
| TRY64 | 2019 | female | Tongren | Yanhe |
| GYY65 | 2020 | male | Guiyang | Yunyan |
| QDZ66 | 2020 | male | Qiandongnan | Zhenyuan |
| ASZ67 | 2019 | male | Anshun | Ziyun |
| GYB68 | 2019 | male | Guiyang | Baiyun |
| QXC69 | 2019 | male | Qianxinan | Ceheng |
| QDC70 | 2021 | male | Qiandongnan | Chengong |
| QDC71 | 2018 | male | Qiandongnan | Congjiang |
| BJD72 | 2019 | male | Bijie | Dafang |
| QND73 | 2021 | male | Qiannan | Dushan |
| QNF74 | 2019 | female | Qiannan | Fuquan |
| QDH75 | 2021 | male | Qiandongnan | Huangping |
| QDK76 | 2021 | female | Qiandongnan | Kaili |
| QNL77 | 2021 | female | Qiannan | Leishan |
| QNL78 | 2020 | male | Qiannan | Libo |
| QNL79 | 2020 | male | Qiannan | Luodian |
| ZYM80 | 2019 | female | Zunyi | Meitan |
| BJN81 | 2019 | male | Bijie | Nayong |
| ASP82 | 2019 | male | Anshun | Pingba |
| QNP83 | 2019 | male | Qiannan | Pingtang |

Table S2. List of primers for VNTR loci amplification

| VNTR Multiplex  PCR  Combination | Locus | Primers Sequence | Modification | Product Length (bp) |
| --- | --- | --- | --- | --- |
| 1 | (AC)8b | GCCCACTTACCTCAACCAAC | VIC | 390 |
|  |  | CCTATAACGGCACTCAGTCCA |  |  |
|  | (GTA)9 | AGCCTTAGTCGCGCAGATG | ROX | 307 |
|  |  | TCCGCTGTCCGTCCGCTGA |  |  |
|  | (GGT)5 | GCAGCGGTGTAACAGCATAGC | 6FAM | 242 |
|  |  | TGTCTGCCTTGCGAAACGGTC |  |  |
|  | (AT)17 | TCTCCAACATGCTGCGACA | PET | 181 |
|  |  | GTACAGCGGCCTGATCGAA |  |  |
|  | rpotT | ATGCCGAACCGGACCTCGACGTTGA | VIC | 91 |
|  |  | TCGTCTTCGAGGTCGTCGAGA |  |  |
| 2 | 21-3 | GAATCTGACCTTTCGGAAATG | 6FAM | 312 |
|  |  | CGATGCAGCTTCCTACGG |  |  |
|  | (AC)9 | AGCGCCCGTTGTCGATAG | ROX | 210 |
|  |  | GACTGGATGTCGGCACCCC |  |  |
|  | (AT)15 | CAATATGCGGGTTGGCGCTTCTG | PET | 168 |
|  |  | CCGTCTGGCTCGATGGCTGGATTC |  |  |
|  | (AC)8a | GTGTTACGCGGAACCAGGCA | VIC | 124 |
|  |  | CCATCTGTTGGTACTACTGA |  |  |
| 3 | 27-5 | ATTGAGCAGATGGCCGGTC | 6FAM | 327 |
|  |  | AGCAGTCGGCACGCCCTT |  |  |
|  | 6-7 | GCCATCGTTGTCGGTTCATC | VIC | 268 |
|  |  | CGGAGGAGGTGGGTACGGT |  |  |
|  | (TA)18 | CGTGCGTCGTGTGTAGGC | ROX | 230 |
|  |  | GACGTGGCAACATCGAAGTT |  |  |
|  | (TTC)21 | GGACCTAAACCATCCCGTTT | PET | 201 |
|  |  | CTACAGGGGGCACTTAGCTC |  |  |
| 4 | 12-5 | CTGGTCCACTTGCGGTACGAC | VIC | 289 |
|  |  | GGAGAAGGAGGCCGAATACA |  |  |
|  | 23-3 | CCGAAGCCCTGGACGAAG | 6FAM | 243 |
|  |  | GCCGTAAATCCGCTCCC |  |  |
|  | 18-8 | GCCCGTCTATCCGCATCAA | PET | 348 |
|  |  | GCAAAGATCAGCACGCCAAT |  |  |
|  | (TA)10 | TAGATTCAAACGACCATGCA | PET | 185 |
|  |  | TGATAATCACGTGTTTCCGC |  |  |

Table S3. Primers for classification SNP

| Locus position | Primers Sequence |
| --- | --- |
| 14,676 (L1) | AATGGAATGCTGGTGAGAGC |
|  | CAATGCATGCTAGCCTTAATGA |
| 1,642,875 (L2) | CTCGTCACAAATCCGAGTTTGAAT |
|  | GTAGTAGTCTTCCAAGTTGTGGTG |
| 2,935,685 (L3) | ATCTGGTCCGGGTAGGAATC |
|  | ACCGGTGAGCGCACTAAG |
| SNP-8,453 | GGTCTGCGGACAAGTTGGTA |
|  | CAATAGCGCTCAGACACGAC |
| SNP-313,361 | CACCGGAGACAAAGCTGAT |
|  | CTCGGAGACCAAACTTCTCG |
| SNP-61,425 | TCGTCAAGCCGAAAGAGTTT |
|  | CCAGAACACCGAGGGAATAA |
| SNP-1,642,875 | TTGAATGCGACCAAACGTACTTTCTG |
|  | TACCACCGGATCATGGAACCGTC |
| SNP-1,133,492 | GCCAAATATAATCTTGCACAGG |
|  | CCCGGGTTTGTCTCCTAACT |
| SNP-2,312,059 | TCAGAGTTTCCGGATGAACC |
|  | CGAGCTAGCCGTCAAAAGG |
| SNP-413,902 | ATCCGGTGCTGGAGTGTCT |
|  | GGTGGTACCTGTAGCGCAAT |
| SNP-20,910 | CAGGTGAACTTCGGACATGC |
|  | CGAGTCAACTGGACGAATCA |
| SNP-1,079,902 | CTTTTGGCAATATCGCAATG |
|  | ACGACCGCAAGAATCTCAGT |

Table S4. VNTR profiles and allele diversity of the 17 polymorphic loci.

| copy number | VNTR loci | | | | | | | | | | | | | | | | |  |
| --- | --- | --- | --- | --- | --- | --- | --- | --- | --- | --- | --- | --- | --- | --- | --- | --- | --- | --- |
|  | (AC)  8b | (GTA)9 | (GGT)5 | (AT)17 | rpoT | 21-3 | (AC)  9 | (AT)  15 | (AC)  8a | 27-5 | 6-7 | (TA)  18 | (TTC)  21 | 18-8 | 12-5 | 23-3 | (TA)  10 | |
| 1 |  |  |  |  |  | 1 |  |  |  |  |  |  |  |  |  |  |  | |
| 2 |  |  |  |  |  | 82 |  |  |  |  |  |  |  |  | 2 | 81 |  | |
| 3 |  |  | 2 |  | 82 |  |  |  |  |  |  |  |  |  | 81 | 2 |  | |
| 4 |  |  | 80 |  | 1 |  |  |  |  | 1 |  |  |  |  |  |  |  | |
| 5 |  |  | 1 |  |  |  |  |  |  | 75 |  |  |  |  |  |  |  | |
| 6 |  |  |  |  |  |  |  |  |  | 7 |  |  |  | 4 |  |  |  | |
| 7 | 14 |  |  |  |  |  | 6 |  |  |  | 12 |  |  | 75 |  |  |  | |
| 8 | 55 |  |  |  |  |  | 56 |  | 6 |  | 44 |  |  | 3 |  |  | 4 | |
| 9 | 12 |  |  |  |  |  | 17 |  | 48 |  | 19 |  |  | 1 |  |  | 12 | |
| 10 | 1 |  |  | 14 |  |  | 3 |  | 12 |  | 6 |  | 3 |  |  |  | 14 | |
| 11 | 1 | 1 |  |  |  |  | 1 |  | 9 |  | 1 |  |  |  |  |  | 17 | |
| 12 |  | 1 |  |  |  |  |  |  | 8 |  | 1 | 3 | 1 |  |  |  | 10 | |
| 13 |  | 2 |  | 37 |  |  |  | 3 |  |  |  | 9 | 8 |  |  |  | 11 | |
| 14 |  | 7 |  | 18 |  |  |  |  |  |  |  | 14 | 11 |  |  |  | 5 | |
| 15 |  | 2 |  | 8 |  |  |  | 38 |  |  |  | 10 | 7 |  |  |  | 3 | |
| 16 |  | 12 |  | 5 |  |  |  | 9 |  |  |  | 10 | 11 |  |  |  | 2 | |
| 17 |  | 8 |  | 1 |  |  |  | 12 |  |  |  | 9 | 6 |  |  |  | 1 | |
| 18 |  | 5 |  |  |  |  |  | 4 |  |  |  | 3 | 4 |  |  |  | 1 | |
| 19 |  | 3 |  |  |  |  |  |  |  |  |  | 2 | 1 |  |  |  | 1 | |
| 20 |  | 6 |  |  |  |  |  | 2 |  |  |  | 8 | 1 |  |  |  | 1 | |
| 21 |  | 9 |  |  |  |  |  | 5 |  |  |  | 3 | 4 |  |  |  | 1 | |
| 22 |  | 3 |  |  |  |  |  | 2 |  |  |  |  | 1 |  |  |  |  | |
| 23 |  | 2 |  |  |  |  |  | 4 |  |  |  | 2 | 4 |  |  |  |  | |
| 24 |  | 6 |  |  |  |  |  | 2 |  |  |  | 2 | 6 |  |  |  |  | |
| 25 |  | 4 |  |  |  |  |  | 1 |  |  |  | 2 | 2 |  |  |  |  | |
| 26 |  | 5 |  |  |  |  |  | 1 |  |  |  | 6 | 2 |  |  |  |  | |
| 27 |  | 1 |  |  |  |  |  |  |  |  |  |  | 1 |  |  |  |  | |
| 28 |  | 1 |  |  |  |  |  |  |  |  |  |  | 1 |  |  |  |  | |
| 29 |  |  |  |  |  |  |  |  |  |  |  |  | 1 |  |  |  |  | |
| 30 |  | 2 |  |  |  |  |  |  |  |  |  |  | 2 |  |  |  |  | |
| 31 |  |  |  |  |  |  |  |  |  |  |  |  | 1 |  |  |  |  | |
| 32 |  | 1 |  |  |  |  |  |  |  |  |  |  |  |  |  |  |  | |
| 33 |  |  |  |  |  |  |  |  |  |  |  |  | 3 |  |  |  |  | |
| 34 |  |  |  |  |  |  |  |  |  |  |  |  | 1 |  |  |  |  | |
| 35 |  |  |  |  |  |  |  |  |  |  |  |  | 1 |  |  |  |  | |
| HGDI | 0.518 | 0.933 | 0.071 | 0.721 | 0.024 | 0.024 | 0.502 | 0.755 | 0.626 | 0.178 | 0.648 | 0.909 | 0.937 | 0.182 | 0.048 | 0.048 | 0.879 | |

Figure S1. Schematic of M. leprae SNP subtyping of clinical leprosy samples based on PCR-RFLP.


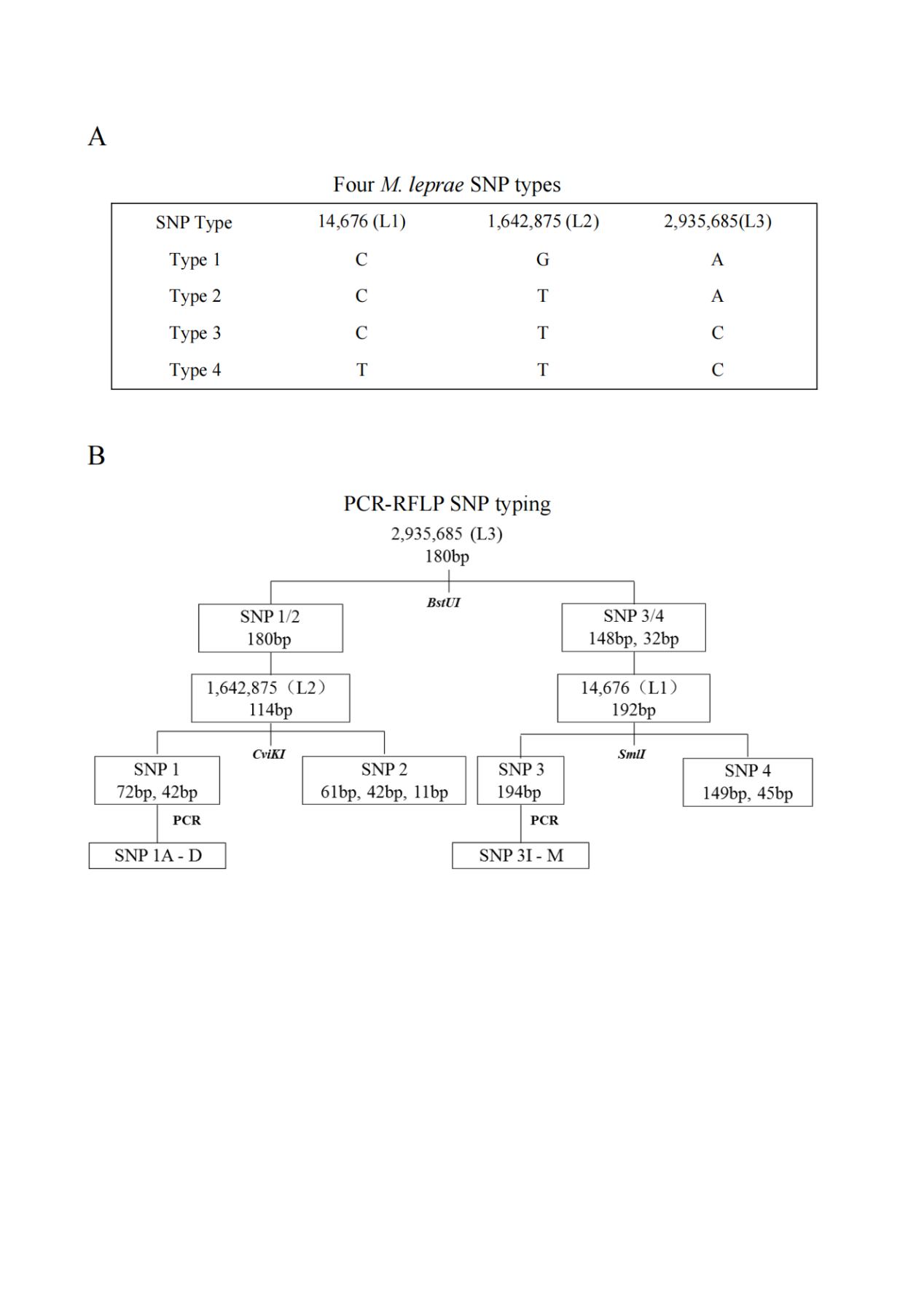


1. The four major SNP types. The numbers below the SNP loci refer to the nucleotide positions in the sequenced TN strain. (B) Scheme of M. leprae SNP subtyping.

Figure S2. Position and nucleotide variation for SNP subtyping


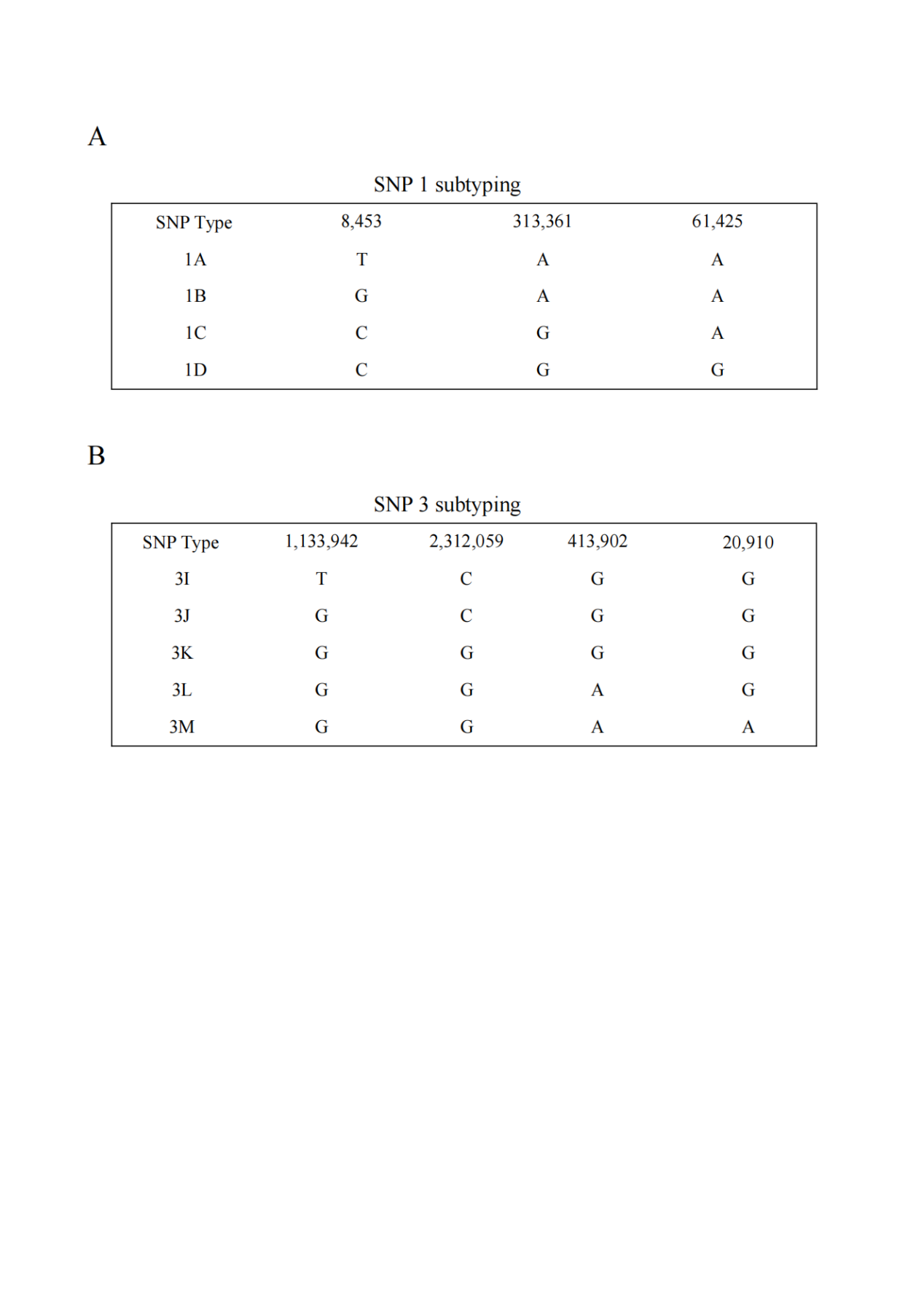


Figure S3. Population structure and clustering pattern


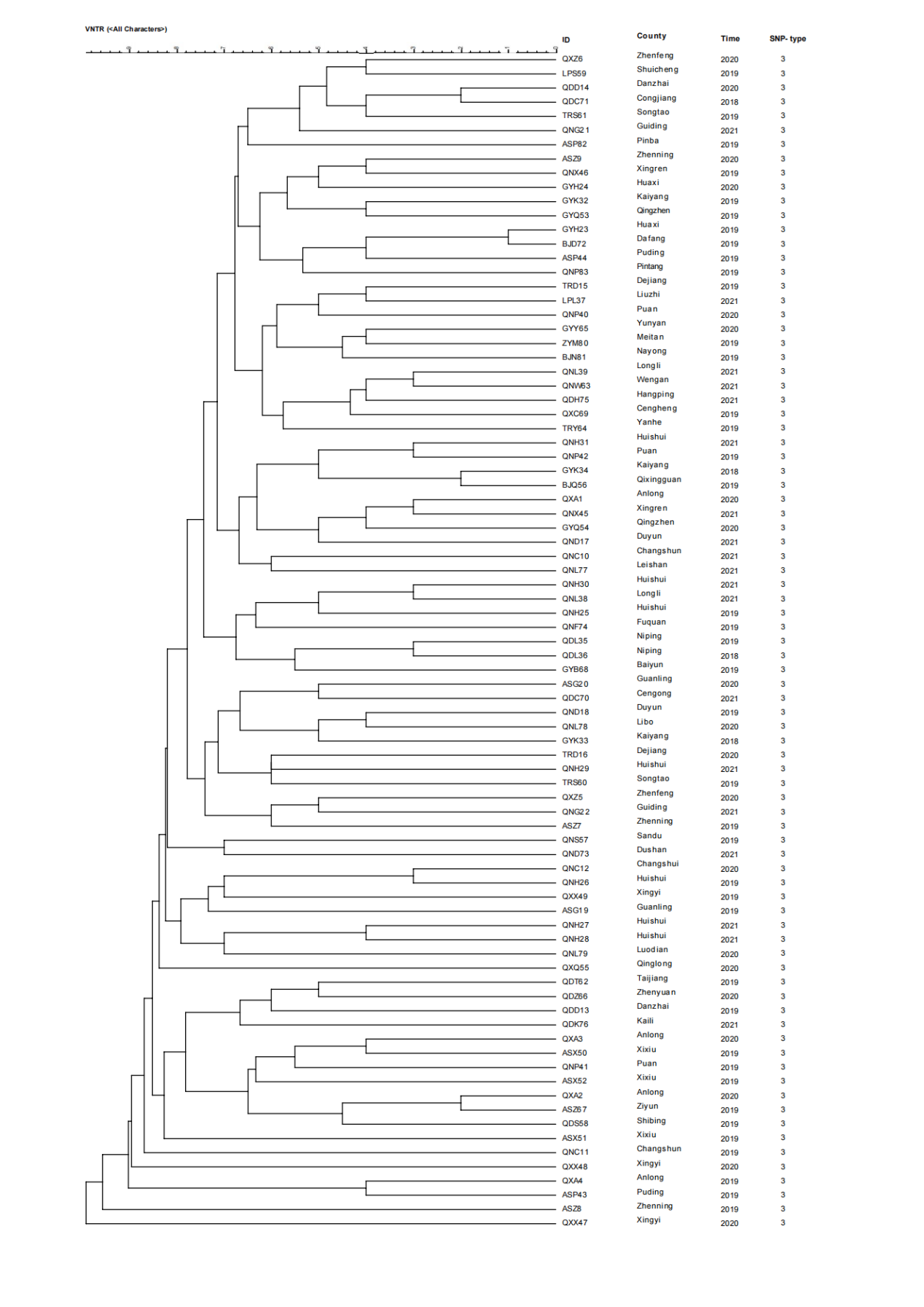

Supplement: Supplementary file 1 [file Data_Sheet_1.docx]
